# Performance of Fetal Medicine Foundation Software for Pre-Eclampsia Prediction Upon Marker Customization: Cross-Sectional Study

**DOI:** 10.2196/14738

**Published:** 2019-11-22

**Authors:** Karina Bilda De Castro Rezende, Antonio José Ledo Alves Cunha, Joffre Amim Jr, Wescule De Moraes Oliveira, Maria Eduarda Belloti Leão, Mariana Oliveira Alves Menezes, Ana Alice Marques Ferraz De Andrade Jardim, Rita Guérios Bornia

**Affiliations:** 1 Programa de Pós Graduação em Clínica Médica Faculdade de Medicina Universidade Federal do Rio de Janeiro Rio de Janeiro Brazil; 2 Maternidade Escola Universidade Federal do Rio de Janeiro Rio de Janeiro Brazil; 3 Programa de Mestrado Profissional em Saúde Perinatal Maternidade Escola Universidade Federal do Rio de Janeiro Rio de Janeiro Brazil; 4 Laboratório Multidisciplinar de Epidemiologia e Saúde -LAMPES Universidade Federal do Rio de Janeiro Rio de Janeiro Brazil; 5 Faculdade de Medicina Universidade Federal do Rio de Janeiro Rio de Janeiro Brazil

**Keywords:** decision support techniques, mass screening, pre-eclampsia, ethnicity, algorithms

## Abstract

**Background:**

FMF2012 is an algorithm developed by the Fetal Medicine Foundation (FMF) to predict pre-eclampsia on the basis of maternal characteristics combined with biophysical and biochemical markers. Afro-Caribbean ethnicity is the second risk factor, in magnitude, found in populations tested by FMF, which was not confirmed in a Brazilian setting.

**Objective:**

This study aimed to analyze the performance of pre-eclampsia prediction software by customization of maternal ethnicity.

**Methods:**

This was a cross-sectional observational study, with secondary evaluation of data from FMF first trimester screening tests of singleton pregnancies. Risk scores were calculated from maternal characteristics and biophysical markers, and they were presented as the risk for early pre-eclampsia (PE34) and preterm pre-eclampsia (PE37). The following steps were followed: (1) identification of women characterized as black ethnicity; (2) calculation of early and preterm pre-eclampsia risk, reclassifying them as white, which generated a new score; (3) comparison of the proportions of women categorized as high risk between the original and new scores; (4) construction of the receiver operator characteristic curve; (5) calculation of the area under the curve, sensitivity, and false positive rate; and (6) comparison of the area under the curve, sensitivity, and false positive rate of the original with the new risk by chi-square test.

**Results:**

A total of 1531 cases were included in the final sample, with 219 out of 1531 cases (14.30; 95% CI 12.5-16.0) and 182 out of 1531 cases (11.88%; 95% CI 10.3-13.5) classified as high risk for pre-eclampsia development, originally and after recalculating the new risk, respectively. The comparison of FMF2012 predictive model performance between the originally estimated risks and the estimated new risks showed that the difference was not significant for sensitivity and area under the curve, but it was significant for false positive rate.

**Conclusions:**

We conclude that black ethnicity classification of Brazilian pregnant women by the FMF2012 algorithm increases the false positive rate. Suppressing ethnicity effect did not improve the test sensitivity. By modifying demographic characteristics, it is possible to improve some performance aspects of clinical prediction tests.

## Introduction

### Pre-Eclampsia

Pre-eclampsia is predominant in gestational hypertensive disorders, with a significant impact on maternal and neonatal health [1,2]. Many researchers aim to identify pre-eclampsia development in high-risk pregnancies by using an effective predictive model. This would allow the implementation of strategies for efficient prevention of disease occurrence in a selected population, thereby reducing its prevalence [3]. At present, there is no customized model for clinical use in pregnant Brazilian women.

### The Fetal Medicine Foundation Software

FMF2012 [4] is an algorithm, developed by the Fetal Medicine Foundation (FMF) to predict pre-eclampsia on the basis of maternal characteristics combined with biophysical and biochemical markers is available on the FMF website at no cost [5]. It estimates the likelihood of developing pre-eclampsia from maternal factors (ethnicity/skin color, age, weight, height, history of diabetes, chronic hypertension, autoimmune diseases, and use of assisted reproduction techniques), together with biophysical markers such as mean arterial pressure and uterine artery pulsatility index (UtAPI) [5,6]. The objective of screening in the first trimester is to identify women at high risk for preterm pre-eclampsia (<37 weeks) and reduce such a risk through prophylactic use of low-dose aspirin.

### Brazilian Experience

In 2016, this FMF2012 model was tested in a sample of pregnant Brazilian women, and its performance was found to be unsatisfactory because of differences in the contribution of risk factors such as ethnicity/skin color [7,8]. According to the FMF, the screening-positive rate in black women is greater than that in white women, as an inevitable consequence of the fact that the prevalence of preterm pre-eclampsia is more than three time higher in black than in white women [9].

Afro-Caribbean ethnicity is the second most common risk factor identified in populations tested by the FMF [10], which was not confirmed in our population. The variable, maternal ethnicity, applied in the FMF2012 algorithm overestimates the risk, which compromises the performance of the screening, as it is a variable with a coefficient of great magnitude [7].

We proposed to suppress the effect of ethnicity on the risk estimated for pre-eclampsia in the sample studied.

### Objectives

The objective of this study was to analyze the performance of pre-eclampsia prediction software by customization of maternal ethnicity in a Brazilian scenario.

## Methods

### Study Design

This was a cross-sectional observational study, with secondary evaluation of data from first trimester screening tests of single-fetus pregnancies performed between October 2010 and December 2015.

### Setting

The study was conducted at the Maternidade Escola da Universidade Federal do Rio de Janeiro, a nonprofit university hospital that exclusively serves patients from the public health system and receives undergraduate and postgraduate students in the health care sector. The local ethics committee approved the study protocol (CAAE: 78764117.0.0000.5275).

The following exclusion criteria were the same as described by the FMF and applied to the original study [8]: pregnancy with chromosomal or structural abnormality, miscarriage or fetal death before 24 weeks of gestation, use of acetylsalicylic acid (ASA) during pregnancy before 16 weeks of gestation, and delivery of a small-for-gestational-age newborn to a mother without pre-eclampsia.

### First Trimester Screening Scan

Patients were scheduled for a first trimester screening scan at 11+0 to 13+6 weeks of gestation. This examination included recording of maternal characteristics, measurement of fetal crown-rump length, measurement of right and left UtAPIs by transabdominal color Doppler ultrasound (Nemio, Toshiba; Xario, Toshiba; Medison V10, Medison; or Aloka, Aloka Co), and measurement of mean arterial pressure with an automated device (3BTO-A2, Microlife or OMRON, OMRON Corporation) by using a standardized method (in both arms simultaneously, while the mother was sitting after ≥10-min rest) [11]. All data were entered into the FMF2012 software.

In the FMF software, the lists in risk calculation for ethnicity classification have been fixed, and the mother should be categorized using the following [5]: (1) white (European, Middle Eastern, North African, and Hispanic), (2) black (African, Caribbean, and African American), (3) East Asian (Chinese, Japanese, and Korean), (4) South Asian (Indian, Pakistani, and Bangladeshi), (5) mixed (white-black, white–East Asian, white–South Asian, black–East Asian, black–South Asian, and East Asian–South Asian). However, in Brazil and in our study, the criterion to define ethnicity was self-qualification of skin color [1,12].

The maternal characteristics were collected from a patient questionnaire administered by a medical doctor. Continuous variables were maternal age (in years), weight (in kilograms), and height (in centimeters). Categorical variables were self-reported ethnicity (black, white, yellow, indigenous, or mixed) [12], parity (nulliparous, parous with no previous pre-eclampsia, or parous with previous pre-eclampsia), maternal family history of pre-eclampsia (yes or no), smoking during pregnancy (yes or no), history of previous hypertension (yes or no), type 1 diabetes (yes or no), type 2 diabetes (yes or no), systemic lupus erythematosus or antiphospholipid syndrome (yes or no), and use of assisted reproductive technology (yes or no).

The biophysical markers considered in this study were crown-rump length (in millimeters), mean arterial pressure (in mm Hg and multiples of median) [11], and mean UtAPIs (arithmetic mean and in multiples of median) [13]. The FMF2012 algorithm calculated multiples of median values by using a multiples of median equation [6].

Risk scores were calculated according to the competitive risk model described by Wright et al [6] from maternal characteristics and biomarkers (mean arterial pressure and UtAPIs), and these were presented as the risk of pre-eclampsia development before 34 and 37 weeks. The cut-off values for positivity for these timepoints were 1/200 and 1/57, respectively [14].

Data on pregnancy outcomes (pre-eclampsia occurrence and gestational age at delivery) were collected from hospital records. The diagnosis of early pre-eclampsia was based on the onset of systolic blood pressure≥140 mm Hg or diastolic blood pressure≥90 mm Hg and proteinuria (protein excretion>300 mg/24 hours after 20 weeks of gestation), which requires delivery before 34 weeks (pre-eclampsia<34 weeks or early pre-eclampsia) or before 37 weeks (pre-eclampsia<37 weeks or preterm pre-eclampsia) [14,15]. Gestational age at birth was calculated on the basis of the last menstrual period or first trimester ultrasound screening. When the difference between these timepoints was >7 days, ultrasound estimation was used.

For our purpose, the following process was undertaken: (1) identification of pregnant women characterized as ethnically black from recalled first trimester reports; (2) calculation of risk of early pre-eclampsia and preterm pre-eclampsia by the FMF2012 algorithm in these pregnant women, reclassifying them as white, which generates a new score; (3) comparison of the proportion of women categorized as high risk by the original and new scores; (4) construction of the receiver operator characteristic curve; (5) calculation of the area under the curve (AUC), sensitivity, and false-positive rate (FPR) and respective 95% CIs; and (6) comparison of the AUC, sensitivity, and FPR of the original risk with the *new* risks by using a Chi-square test (the differences were considered significant if *P*<.05). STATA 13 statistical software package (StataCorp, College Station, Texas) was used for data analyses.

## Results

First trimester screening was carried out in 1934 singleton pregnancies. We excluded 403 cases because of fetal aneuploidies (n=7); major fetal malformation (n=28); miscarriage, termination, or fetal death before 24 weeks of gestation (n=18); ASA use at ≤16 weeks of gestation (n=103); small-for-gestational-age neonatal status in the absence of pre-eclampsia (n=69); and missing outcome data (n=178). The remaining 1531 cases were included in the study. We identified 645 (645/1531, 42.12%) patients classified as mixed, 589 (589/1531, 38.47%) as white, and 296 (296/1531, 19.33%) as ethnically black. The sample presented 11 (0.71%) cases of early pre-eclampsia and 26 (1.69%) cases of preterm pre-eclampsia. We observed that 3 of 11 cases (27%) of early pre-eclampsia and 6 of 26 (23%) cases of preterm pre-eclampsia occurred in pregnant women primarily classified as ethnically black.

Mean maternal weight, height, and age were 67 kg, 160 cm, and 27 years, respectively. According to the predetermined cut-off values, 219 of 1531 cases (14.30%, 95% CI 12.5-16.0) of our final sample were classified to be at high risk of pre-eclampsia development. After we recalculated the *new risk*, 182 of 1531 cases (11.88%, 95% CI 10.3-13.5) of the final sample were categorized as being at high risk of pre-eclampsia development.

The pre-eclampsia rate in this sample was not different in relation to ethnicity, smoking, family history of pre-eclampsia, or assisted reproductive technology use; as our sample contained few cases of the latter, significant inference was not drawn in this case. In addition, no case of systemic lupus erythematosus or antiphospholipid syndrome was detected in our sample.

Table 1 presents an evaluation of the performance of the FMF2012 predictive model among the studied population, according to the originally estimated risks, with pregnant black women classified as ethnically black, and the *newly estimated risks*, which consider all patients as white/mixed race (baseline risk) for pre-eclampsia<34 weeks and pre-eclampsia<37 weeks. The comparison of the FMF2012 predictive model performance between the originally estimated risks and the *newly estimated risks* showed that the difference was not significant for sensitivity, but it was significant for FPR.

**Figure 1:**
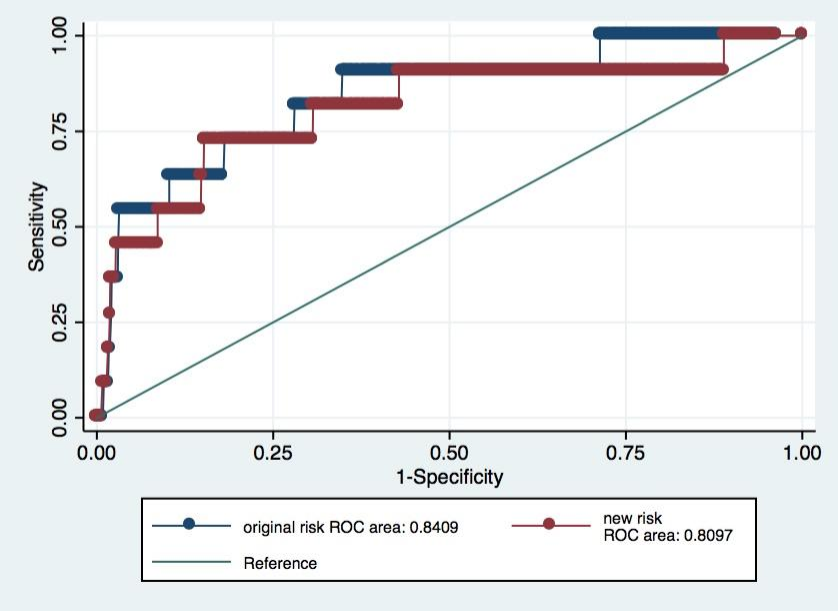
Receiver operator characteristic curves of the original risk and new risk for pre-eclampsia <34. ROC: receiver operator characteristic.

**Figure 2:**
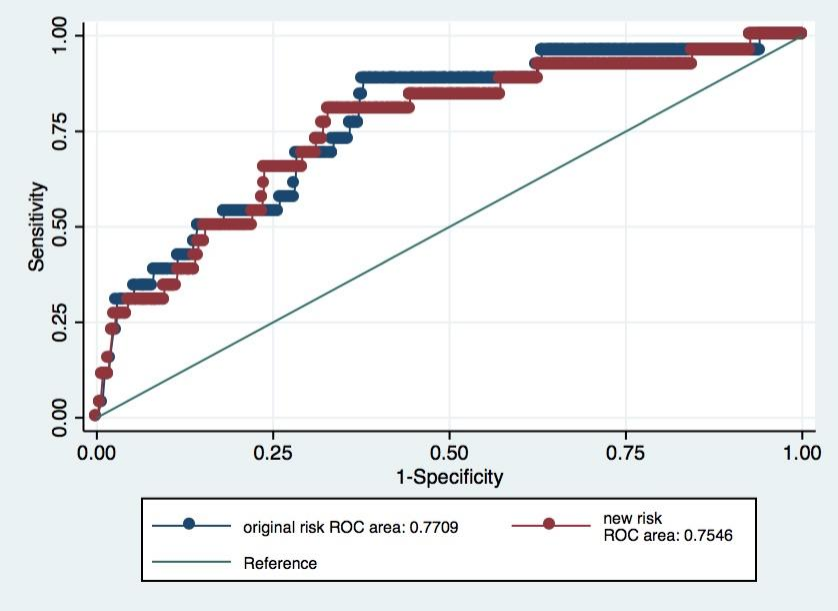
Receiver operator characteristic curves of the original risk and new risk for pre-eclampsia <37. ROC: receiver operator characteristic.

Figures 1 and 2 present the AUC and the comparison between receiver operator characteristic curves of the original risk and *new risk* for pre-eclampsia<34 weeks and pre-eclampsia<37 weeks, respectively. There were no significant differences between the curves.

**Table 1:** Results of the evaluation of the performance of the Fetal Medicine Foundation 2012 (FMF2012) predictive model.

Outcome		Sensitivity (%), (95% CI)	*P* value	False-positive (%), (95% CI)	*P* value	Area under curve (95% CI)	*P* value
**Pre-eclampsia** **<34 weeks**							
	Original risk	63 (35-92)	.66	13.9 (12.2-15.6)	.05^a^	0.84 (0.71-0.97)	.17
	New risk	54 (25-89)	.66	11.5 (10-13)	.05^a^	0.80 (0.65-0.96)	.17
**Pre-eclampsia** **<37 weeks**							
	Original risk	46 (27-65)	.57	13.9 (12.2-15.7)	.04^a^	0.77 (0.68-0.86)	.36
	New risk	38 (20-57)	.57	11.5 (10-13)	.04^a^	0.76 (0.65-0.85)	.36

^a^*P*≤.05.

## Discussion

### Principal Findings

This study proposed a new strategy to use the predictive model of pre-eclampsia, FMF2012, in the first trimester of gestation in Brazilian women, to optimize its application; a customized model for our population is not available as yet. However, pre-eclampsia prediction was not improved by the suggested strategy, as the sensitivity remained the same. A simulation that all pregnant women who were submitted to pre-eclampsia screening in the first trimester, along the study period, would belong to the white race was applied, which represents the baseline risk of the predictive model, suppressing the effect of race on the model. It was found that a significantly lower proportion of screened pregnant women would be categorized as high risk with this approach, which implies a reduction in the FPR.

According to the FMF2012 algorithm for pre-eclampsia risk assessment, maternal racial origin is a categorical variable with the following possible values: *white*, *black*, *East Asian*, *South Asian*, or *mixed*; only black and South Asian ethnicity demonstrate significant contribution for the prediction of pre-eclampsia [6,16,17]. 

Ethnic disparities remain to be a contentious matter [18]. The racial classification applied in our country by Instituto Brasileiro de Geografia e Estatística identifies people with regard to their race, and it is also used in national administrative databases [19]. Biological methods based on the identification of biogeographical ancestry are not suited for the intended purposes, and the racial composition obtained by self-classification seems to be the most accurate because of historical and theoretical reasons [20]. 

In this study, the criterion to define ethnicity was self-qualification of skin color, which constitutes one of the characteristics that comprises ethnicity that is not associated with ancestry, which, in turn, contributes to the validation of this attribute in the model, as it is not the racial determinant, especially in the mixed Brazilian population [20]. The reclassification of black patients as ethnically white creates a new score that is less than the original score, as being ethnically white denotes the baseline risk and eliminates the differences regarding the risk of pre-eclampsia development. FMF published that the prevalence of preterm pre-eclampsia is more than three times higher in black than in white women, which was not observed in our sample, which results in a greater FPR.

Even with a small number of early pre-eclampsia cases in the studied sample, a significant decrease in the FPR was quantified. However, there was no significant difference in the sensitivity and AUC, which dictates the performance of diagnostic tests. This was also observed in the performance of preterm pre-eclampsia screening, but the number of cases was twice as that in this study. Although, in population terms, the improvement in the FPR was small and the study did not have a considerable impact on overall detection rates, making allowances for ethnic origin can make a significant difference to an individual patient-specific risk, which could alter clinical decision making, mainly with regard to ASA prescription [21,22]. Furthermore, incorrect classifications of a pregnant woman as high risk could cause her to follow centralized prenatal care with frequent exams and rigorous protocols that could be stressful and adversely impact the financial and social costs.

The limitations of this study are related to the small number of cases of early and preterm pre-eclampsia in the sample and mainly in ethnically black women, and this study addressed only one maternal characteristic included as a predictor factor of pre-eclampsia. This study focused on a regional question regarding the performance of the FMF2012 algorithm in pre-eclampsia screening. Although Brazil is a country of continental dimensions with widespread and social inequalities, there are some islands of quality health assistance that can create effective screening strategies and disseminate them by using training programs.

### Conclusions

This study makes an important contribution to the understanding of the effect of black ethnicity in our sample. It also tests an alternative approach that can improve prenatal follow-up and health indicators. It is a work-around approach to employ and take advantage of available software of clinical prediction models. Users are allowed to customize demographic characteristics to adjust predefined coefficients, in different ways, without changing the algorithm structure. This approach can be extended to other characteristics in other algorithms, but the knowledge of the effect of the subject’s characteristic as a risk factor on the target population is a key pillar to achieve performance improvement in clinical prediction models with the proposed strategy in different scenarios. In our sample, making allowance for ethnic origin can make a significant difference to an individual patient-specific risk, which could alter clinical decision making. In conclusion, the classification of pregnant Brazilian women as ethnically black by an FMF2012 pre-eclampsia screening test increases the FPR. Suppressing the effect of ethnicity did not improve test sensitivity. By modifying demographic characteristics, it is possible to improve some performance aspects of clinical prediction tests.

## Abbreviations

ASA: acetylsalicylic acid
AUC: area under the curve
FMF: Fetal Medicine Foundation
FPR: false-positive rate
UtAPIs: uterine artery pulsatility index
